# Nitrogen-Doped Carbon Coated WS_2_ Nanosheets as Anode for High-Performance Sodium-Ion Batteries

**DOI:** 10.3389/fchem.2018.00236

**Published:** 2018-08-23

**Authors:** Yong Liu, Huijie Wei, Chao Wang, Fei Wang, Haichao Wang, Wanhong Zhang, Xianfu Wang, Chenglin Yan, Bok H. Kim, Fengzhang Ren

**Affiliations:** ^1^The Key Laboratory of Henan Province on Nonferrous Metallic Materials Science and Fabrication Technology, Collaborative Innovation Center of Nonferrous Metals of Henan Province, School of Materials Science and Engineering, Henan University of Science and Technology, Luoyang, China; ^2^Jiangsu Provincial Key Laboratory for Advanced Carbon Materials and Wearable Energy Technologies, Collaborative Innovation Center of Suzhou Nano Science and Technology, College of Physics, Optoelectronics and Energy, Soochow Institute for Energy and Materials Innovations, Soochow University, Suzhou, China; ^3^Division of Advanced Materials Engineering, Hydrogen and Fuel Cell Research Center, Chonbuk National University, Jeonbuk, South Korea

**Keywords:** tungsten disulfide, N-doped carbon, nanosheets, sodium ion batteries, electrochemical performances

## Abstract

Due to the cost-effectiveness of sodium source, sodium-ion batteries (SIBs) have attracted considerable attention. However, SIBs still have some challenges in competing with lithium-ion batteries for practical applications. Particularly, the high rate capability and cycling stability are posing big problems for SIBs. Here, nitrogen-doped carbon-coated WS_2_ nanosheets (WS_2_/NC) were successfully synthesized by a high-temperature solution method, followed by carbonization of polypyrrole. When used as anode electrodes for SIBs, WS_2_/NC composite exhibited high-rate capacity at 386 and 238.1 mAh g^−1^ at 50 and 2,000 mA g^−1^, respectively. Furthermore, even after 400 cycle, the composite electrode could still deliver a capacity of ~180.1 mAh g^−1^ at 1,000 mA g^−1^, corresponding to a capacity loss of 0.09% per cycle. The excellent electrochemical performance could be attributed to the synergistic effect of the highly conductive nature of the nitrogen-doped carbon-coating and WS_2_ nanosheets. Results showed that the WS_2_/NC nanosheets are promising electrode materials for SIBs application.

## Introduction

Nowadays, lithium ion batteries (LIBs) have become the most widely used energy storage devices for many applications ranging from high performance portable electronics and electrical vehicles to sustainable energy smart grids. The advantages of LIBs include high energy density, long life span, and so on (Armand and Tarascon, 2008; Yang et al., 2011; Lu et al., 2017; Wu et al., 2017; Xu et al., 2017; Geng et al., 2018; Wang et al., 2018; Zhang et al., 2018). However, these large-scale applications may be gradually hindered due to insufficient lithium resource and its uneven distribution in the Earth's crust (Hou et al., 2017a; Fu et al., 2018). As a representative of the promising battery systems, sodium-ion batteries (SIBs) have attracted considerable attention as an alternative to LIBs (Hou et al., 2017b; Wei et al., 2017; Zheng et al., 2017; Tang K. et al., 2018). The interest in SIBs comes from the superiority of the sodium element, including its abundance in nature, low price, and negative redox potential (−2.71 V vs. SHE) (Palomares et al., 2012; Li et al., 2013; Slater et al., 2013). However, the practical applications of sodium ion batteries have been hindered by lacking of applicable electrode materials to accommodate sodium ions, which are bigger than Li^+^ in radius (Wang X. et al., 2016). Graphite is well-known for being not suitable to host sodium ions since sodium seldom forms stable intercalation compounds with graphite (Komaba et al., 2011; Tian et al., 2017).

Metal sulfides with layered structures, such as MoS_2_ (Xie et al., 2015), WS_2_ (Wang B. et al., 2016; Wang Y. et al., 2016), SnS (Xiong et al., 2017), VS_2_ (Zhou et al., 2017), and SnS_2_ (Tu et al., 2017), have been investigated as potential anode materials for SIBs (Xie et al., 2015). The layered structure of these types of materials allows sodium ions to intercalate reversibly. However, the further application of two-dimensional metal sulfides is hampered by their intrinsic limitations (Xie et al., 2015). First of all, their intrinsic low electronic conductivity will prevent the fast electrochemically Na^+^ storage. Secondly, these thermally unstable nanomaterials are inclined to restack, due to the high surface energy and interlayer van der Waals attractions (Wang et al., 2014). Furthermore, the remarkable volume expansion during Na^+^ intercalation and deintercalation could lead to poor contact between current collector and active materials and the failure of the electrode, resulting in inferior cycling performances (Xie et al., 2015; Luo et al., 2018).

As one of the promising two-dimensional layered metal sulfides as anode materials for SIBs, WS_2_ has attracted considerable attention in recent years. However, the reversible capacity of the bare WS_2_ is low; the rate performance and long cycling stability of the bare WS_2_ must be improved owing to its poor conductivity and serious aggregation during the insertion/extraction process of sodium-ions into/from WS_2_ layers (Chen et al., 2014). Hence, several efforts were made to improve their electrochemical performances. For example, tungsten disulfides were composited with ordered mesoporous carbon (CMK-3) (Pang et al., 2017), carbon nanotube-reduced graphene oxide (CNT-rGO) (Wang B. et al., 2016), and other carbon materials (Li et al., 2016) as anode materials for SIBs to buffer volume change, and excellent electrochemical performances were obtained. In addition, nitrogen-doped conductive carbon/WS_2_ nanocomposites (WS_2_-NC) were fabricated by doping N element into conductive carbon with WS_2_ nanosheets. The composite electrodes show reversible capacity of ~360 mAh g^−1^ at 100 mA g^−1^, presenting much better electrochemical performances than pristine WS_2_ and WS_2_/conductive carbon (Wang X. et al., 2016).

In this study, we have successfully prepared nitrogen-doped carbon-coated WS_2_ nanosheets (WS_2_/NC) with high content of pyridinic and pyrrolic nitrogen species, and explored their sodium-ion storage performance. The pyridinic and pyrrolic nitrogen species could generate more defects and expose more edge sites in the plane of carbon skeletons, which is beneficial to Na^+^ diffusion. On the other hand, nitrogen-doping could greatly increase the electronic conductivity of the carbon layer coated on the WS_2_ electrode, which delivers faster charge transfer during the Na^+^ intercalation/deintercalation processes. Due to the synergistic effect, the WS_2_/NC composite exhibited superior long-term cycling performance and rate capability than the pure WS_2_ electrode, which may show some potential applications in high performance anode for SIBs.

## Materials and methods

### Synthesis of WS_2_ nanosheets

WS_2_ nanosheets were synthesized by a high-temperature solution method as described elsewhere (Cheng et al., 2014). Typically, WCl_6_ (2 mmol) was mixed with a mixture consisting of 1-octadecene (15 mL) and oleylamine (30 mL) in a flask (100 mL) at ambient temperature. To eliminate water and oxygen, the temperature of the above mixture was firstly increased to 140°C under vigorous stirring in Ar gas atmosphere for about 30 min. Then, the solution was quickly heated to ~300°C and maintained for 30 min under Ar gas protection. Later, a sulfur solution, which consists of sulfur powder (4 mmol) and oleylamine (10 mL), was added into the flask in 10 min at 300°C, and kept for 1 hour. After the mixture was cooled down, WS_2_ nanosheets were obtained by adding absolute ethanol (ca. 40 mL), collected by centrifugation, and washed repetitively with ethanol. After drying for 30 hours by lyophilization to remove the organic residue, the as-made product was annealed in argon at 500°C for 2 h, and WS_2_ nanosheets were obtained later.

### Synthesis of WS_2_/NC

One hundred milligrams of as-prepared WS_2_ nanosheets were first ultrasonically dispersed in 100 mL distilled water to form a suspension, and the obtained suspension was mixed with 0.1 mL pyrrole monomer and 0.01 g FeCl_2_. After addition of 0.5 mL of H_2_O_2_ to the mixture, the mixture was stirred for 6 h. After centrifugation, the product was washed repetitively with distilled water, and vacuum-dried for 12 h. Then, WS_2_/NC nanosheets were obtained, after annealing at 500°C for 2 h under Ar.

### Characterization

The phases of the as-prepared samples were characterized by X-ray diffraction (XRD, Bruker D8 ADVANCE, Cu kα source). The morphology, microstructure, and the nanometer-range energy dispersive X-ray spectroscopy (EDS) were investigated by scanning electron microscopy (SEM, FEI Quanta 200 FEG) and transmission electron microscopy (TEM, JEM-2100F). Raman spectra were recorded on a Raman spectrometer (LabRAM HR Evolution), using an excitation laser wavelength of 532 nm. Surface elemental analysis was performed on an X-ray photoelectron spectroscopy (XPS, Kratos Axis Ultra Dld, Japan).

### Electrochemical measurements

The electrochemical tests were carried out using CR2025-type coin cells. To prepare working electrodes, active materials, super P carbon black, and polyvinylidene fluoride (PVDF) in the weight ratio of 7:2:1 were mixed and dispersed in N-methyl pyrrolidone (NMP) to form slurry. The slurry was uniformly coated on the copper foil and then dried at 80°C overnight in a vacuum oven. The areal loading of active materials on the copper foil was ≈1.0 mg cm^−2^. Coin cells were assembled in an argon-filled glove box with contents of H_2_O and O_2_ below 1 ppm. Sodium foil was used as counter and reference electrode, glass fiber filters (Whatman, 1820-047) were used as separators. The electrolyte was 1 M NaClO_4_ in 1:1 v/v EC/PC. Galvanostatic charge and discharge testing at different specific currents were carried out within 0.01–3.0 V (vs. Na/Na^+^) on a battery testing system (LAND CT 2001A, Wuhan, China) at room temperature. Cyclic voltammetry (CV) and electrochemical impedance spectroscopy (EIS) measurement were conducted on a CHI 660E (Chenhua Shanghai, China) electrochemical workstation.

## Results and discussion

**Figure 1:**
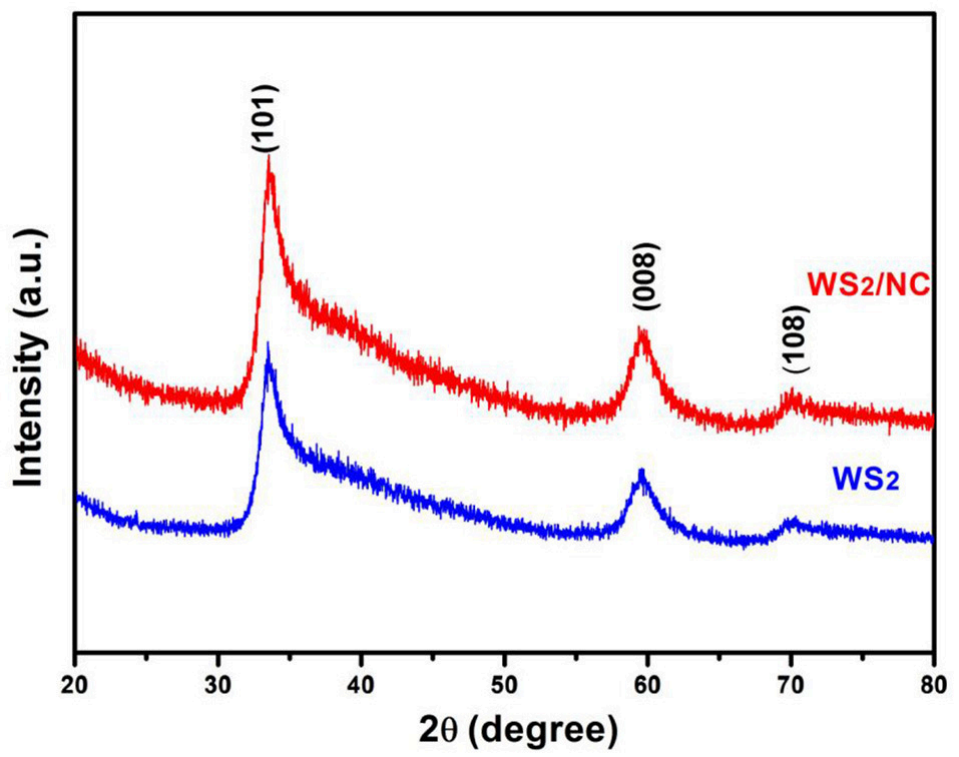
XRD patterns of the as-prepared WS_2_/NC and WS_2_.

The phases of the as-prepared WS_2_ and the WS_2_/NC were analyzed by X-ray diffraction, as shown in Figure 1. The peaks at 33.8°, 59.8°, and 69.9° correspond well to the (101), (008), and (108) planes of the hexagonal structure of WS_2_ (JCPDS No. 84-1398), respectively (Von Lim et al., 2017), indicating successful conversion of the tungsten precursor to layered WS_2_ without any discernible impurities (Cheng et al., 2014). The as-prepared WS_2_/NC sample exhibits the similar XRD pattern as the as-prepared WS_2_, indicating the phase of the WS_2_ remains unchanged after the N-doped carbon coating.

**Figure 2:**
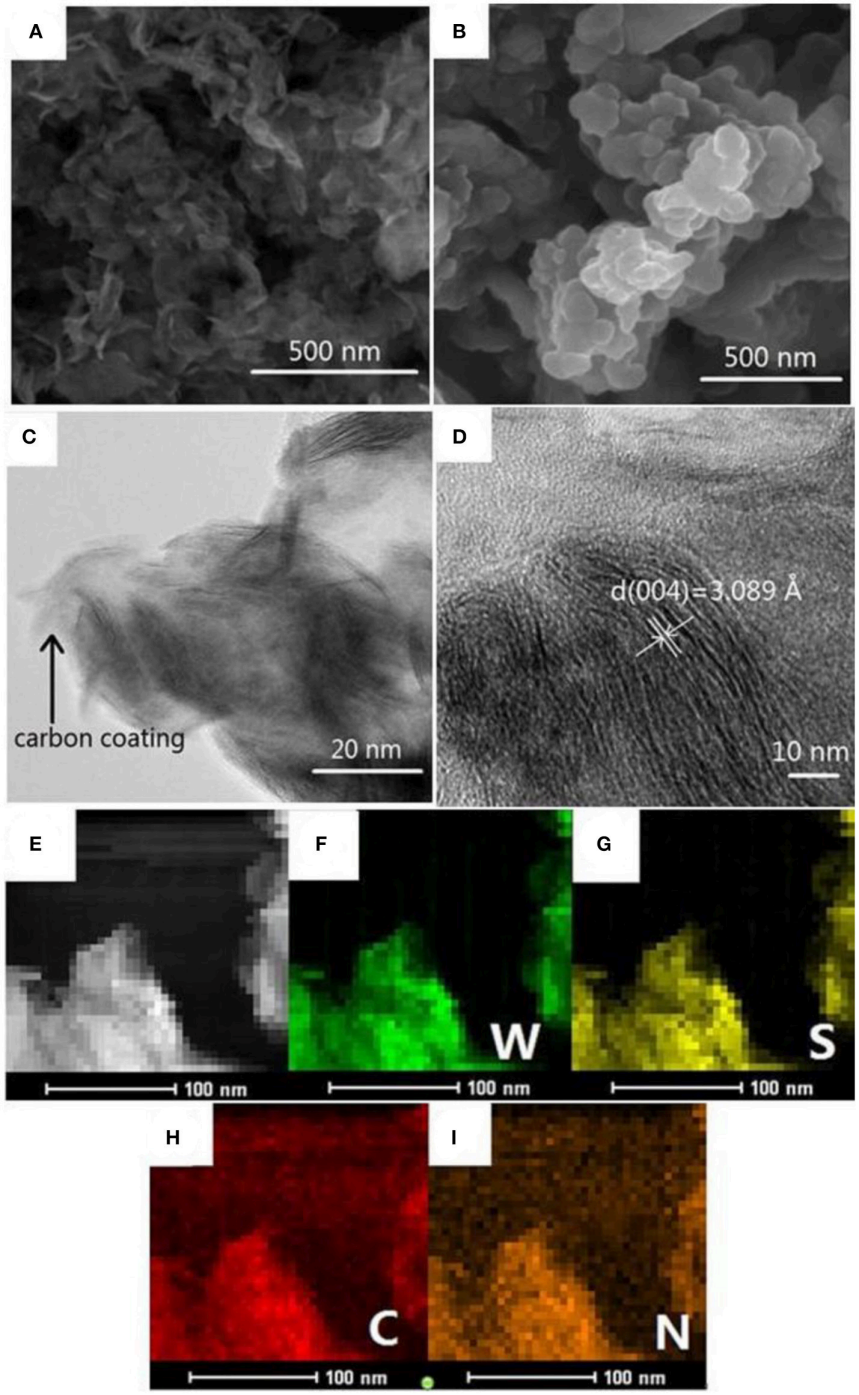
**(A)** SEM image of the WS_2_. **(B)** SEM image of the WS_2_/NC. **(C)** TEM and **(D)** HRTEM images of the WS_2_/NC. **(E)** EDS elemental mapping analysis of the WS_2_/NC and element mapping of **(F)** tungsten, **(G)** sulfur, **(H)** carbon, and **(I)** nitrogen.

The microstructures of the pristine WS_2_ and WS_2_/NC were investigated by SEM and TEM. As displayed in the Figures 2A,B, the WS_2_ and WS_2_/NC nanosheets had typical size of 50-200 nm, demonstrating that as-prepared WS_2_ and WS_2_/NC are nano-size. The SEM images clearly show the flower shape of the WS_2_ nanosheets, with uniform shapes and flat surfaces. The nanometer size can not only shorten the diffusion distance of Na^+^ in charge and discharge process, but also increase the contact between electrode and electrolyte, providing the possibility to improve capacity. From Figure 2B, it can be clearly observed that the morphology of WS_2_/NC is a representative nanosheet. WS_2_/NC nanometer flake has a large surface area and the thickness of atomic scales, which makes WS_2_/NC possess a larger specific surface area, could provide sufficient contact area and could be conducive to the insertion/deinsertion of Na^+^. Further, to investigate the influences of carbon coating to the morphology of WS_2_ nanosheets, we take TEM analysis of WS_2_/NC. As shown in Figure 2C, carbon-derived (pyrrole-carbon) from polypyrrole (PPy) is uniformly coated on WS_2_ nanosheets in nanometer size, and the boundary between WS_2_ and pyrrole-carbon can be clearly distinguished. The carbon-coated structure can greatly improve the electronic conductivity, reduce the resistance of interface, and enhance the diffusion ability of sodium ion. Additionally, the structure can buffer the volume change during the repeated sodiation/desodiation processes and maintain the structural stability of WS_2_ nanosheets, which is beneficial for the rate capability and cycle stability (Li et al., 2017).

In the HRTEM image (Figure 2D) of WS_2_/NC, all the independent nanoplates show clear streaks, which is ascribed to the large interlayer spacing along the c-axis of WS_2_ nanoflakes. As shown in Figure 2D, the lattice fringes with interlayer spacing of 0.3089 nm could be ascribed to (004) planes of hexagonal WS_2_, which is well-adapted to the XRD pattern (JCPDS No. 84-1398). The large spacing between the planes of WS_2_/NC can not only provide a fast path in the process of Na^+^ diffusion but also offer abundant space to store sodium ion, which permits the supply of a higher reversible capacity for the batteries. To further ensure the elements of WS_2_/NC, elemental mapping analysis of the as-prepared materials were carried out. Figures 2E–I demonstrated that the W, S, C, and N elements exist in the WS_2_/NC. The superimposted image of elemental mapping of tungsten to sulfur (Figures 2F,G), and that of carbon to nitrogen (Figures 2H,I) are observed. The results indicate the formation of nitrogen-doped carbon supported on WS_2_ nanosheets, and this is consistent with the HRTEM images.

**Figure 3:**
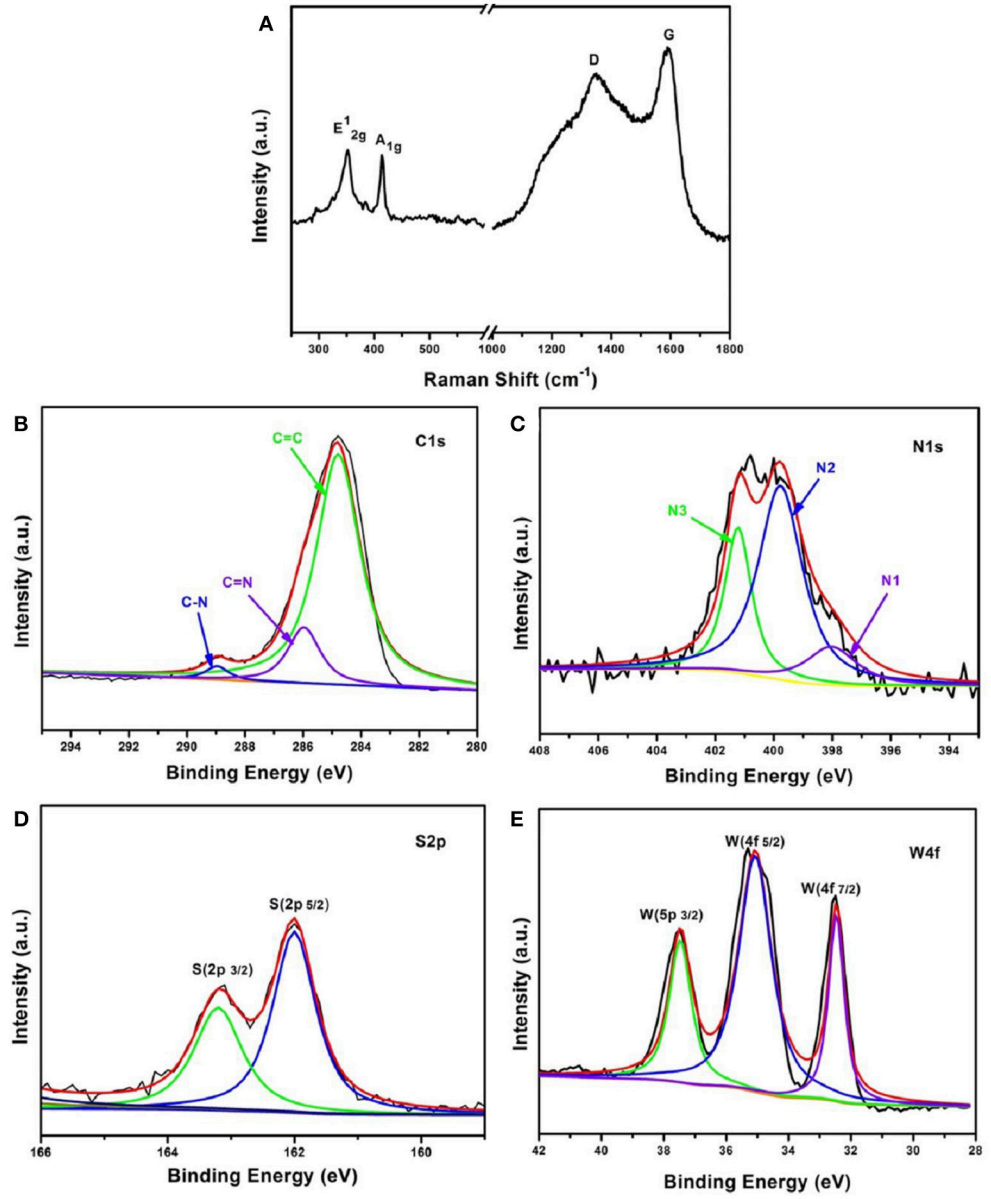
**(A)** Raman spectra and XPS spectra of, **(B)** C 1 s, **(C)** N 1s with N1 (pyridinic-N), N2 (pyrrolic-N), and N3 (graphitic-N), **(D)** S 2p, as well as **(E)** W 4f of the WS_2_/NC samples.

Raman spectra were carried out to further verify the structure of the WS_2_/NC sample. As shown in Figure 3A, two characteristic peaks for WS_2_/NC are located at 350 and 413 cm^−1^, corresponding to the in-plane vibrational mode (E${}_{2\text{g}}^{1}$) and the out-of-plane vibrational mode (A_1g_) of layered WS_2_, respectively (Zeng et al., 2016). In addition, two peaks at 1,350 and 1,585 cm^−1^ can also be observed, which are corresponding to the D and G bands of carbon, and owed to the disordered sp^2^-hybridization of the graphitic carbon structure, and the in-plane vibrational mode of the sp^2^-bonded carbon atoms, respectively (Ferrari et al., 2006). The results are consistent with the morphological studies, indicating that the PPy coated on WS_2_ was partially graphitized after the carbonization process at high temperature.

To make a careful analysis of N-doped carbon coated WS_2_ nanosheets, X-ray photoelectron spectroscopy (XPS) studies were performed. In the C 1 s spectrum of WS_2_/NC (Figure 3B), the sharp peak at about 284.6 eV is associated with sp^2^ carbon with C = C bonds. The other two peaks at around 286 and 288.5 eV correspond to C-N bonds, demonstrating that nitrogen has been successfully incorporated into the carbon layer on WS_2_/NC surface, which is consistent with the Raman results. It is known that nitrogen-doping can efficiently increase the electronic conductivity of the coated carbon layer on the electrode materials, which would lessen the Ohmic polarization and is beneficial for the fast electrochemically ion-storage. N-doping types in the carbon layer was also investigated by XPS as depicted in Figure 3C. The XPS spectrum of N 1 s can be deconvoluted into three main peaks at about 398.7, 401.1, and 403.4 eV, which can be ascribed to pyridinic nitrogen (N1), pyrrolic nitrogen (N2), and graphitic nitrogen (N3), respectively. The graphitic nitrogen is formed by substituting a carbon atom with N atom that only occurred on the edges or inside of the carbon layer, which cannot damage the carbon skeletons (Wang et al., 2012). However, the pyridinic nitrogen is often formed through substituting a carbon atom by N on edges or defect sites in the plane, and the pyrrolic nitrogen species commonly expose planar edges or defect sites, meaning that pyridinic and pyrrolic nitrogen species will cause some defects and more edges in the carbon layer, which could enhance the Na^+^ diffusion velocity, and thus improve the performance of Na^+^ storage (Gao et al., 2015, 2017; Shen et al., 2015). By integrating the area that the fitted curve covered, the relative atomic content of pyridinic and pyrrolic nitrogen species on the surface of the carbon layer was calculated to be above 70%. Such high content of pyridinic and pyrrolic nitrogen species is expected to greatly improve the Na^+^ storage performance of the composite WS_2_/NC electrode. Analysis of the S and W XPS spectra was also performed to study the composition of the WS_2_ in the composite electrode. As shown in Figure 3D, two strong peaks at about 162 and 163.3 eV can be observed, which are associated with the S 2p_5/2_ and S 2p_3/2_ in the WS_2_, respectively. For the XPS spectrum of W represented in Figure 3E, two peaks shown at 32.3 and 34.5 eV corresponding to W 4f_7/2_ and W 4f_5/2_ orbitals, respectively, are in good agreement with the binding energies of W^4+^ in WS_2_ (Tang Y. et al., 2018). Besides, the intensity of peak at 37.8 eV could be assigned to W^4+^ 5p_3/2_. The results demonstrate that no oxidation of W occurred during the preparation process because no W^6+^ signal was found from the XPS results. From the XPS analysis, we can conclude that WS_2_/NC composite with high content of pyridinic and pyrrolic nitrogen species (high defects and edges) in the carbon layer was successfully obtained, which is expect to be a good electrode for Na^+^ storage when used as the anode for Na-ion batteries.

**Figure 4:**
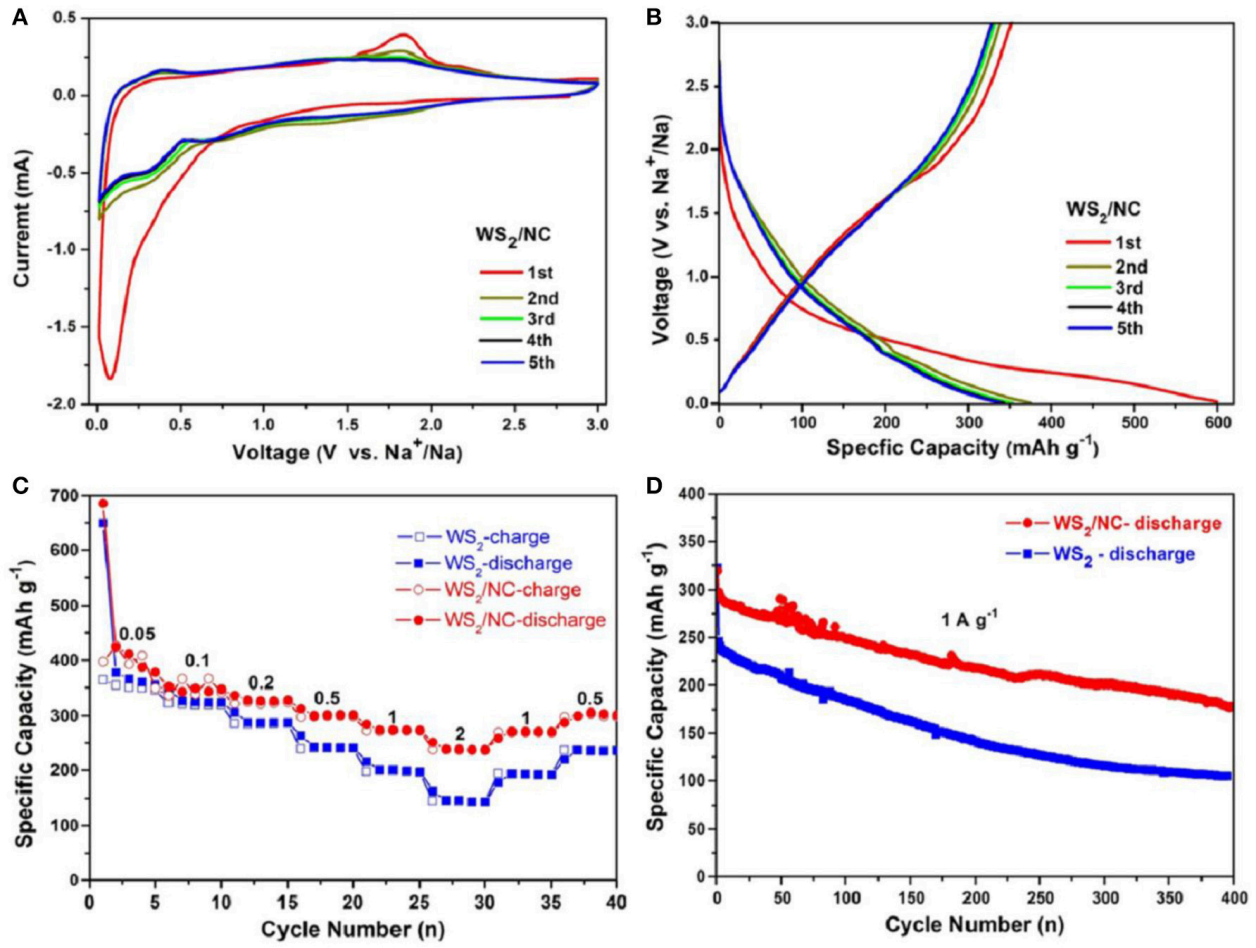
**(A)** CV curves of 1st to 5th cycles of the WS_2_/NC measured at voltage window of 0.01–3.00 V. **(B)** Galvanostatic charge/discharge profiles of the 1st to 5th cycles of WS_2_/NC measured at 200 mA g^−1^. **(C)** Galvanostatic rate capabilities of WS_2_/NC and pure WS_2_ electrodes measured at different current densities. **(D)** Cycling performance of WS_2_/NC and pure WS_2_ electrodes measured at 1 A g^−1^.

Sodium-ion storage performance of the composite electrode was investigated in coin cells using Na metal as the counter electrode, and 1 M NaClO_4_ in EC/PC as electrolyte. The sodiation and desodiation of WS_2_/NC composite anode was firstly characterized using cyclic voltammetry (CV) in the range of voltage is 0.01~3.0 V vs. Na^+^/Na with a scan rate of 0.5 mV s^−1^. As shown in Figure 4A, there are two obviously reductive peaks at 0.08 and 0.36 V, corresponding to the formation of the solid electrolyte interface (SEI) layer and the insertion of Na^+^ in the first discharge process, during which, the Na^+^ was firstly inserted into the crystal of WS_2_ and formed Na_x_WS_2_ without phase transformation. At the low potential of 0.08 V, metal tungsten and Na_2_S were formed by the conversion reaction of WS_2_ crystal and sodium ions. During the charge process, the reversible process can occur, and the peak at 1.88 V is associated with the oxidation of Na_x_WS_2_ into tungsten disulfide in the process of desodiation. In the second cycle, the peaks of 0.47 and 1.88 V in the charge process and the peaks of 0.38 and 0.74 V in the discharge process ascribe to the formation of different chemical compound, respectively. Along with the increase of cycle number, the CV curves turn gradually steady. The two curves of the fourth and the fifth cycle basically overlap, demonstrating the reversible intercalation/deintercalation of sodium ion into/from WS_2_ is more and more stable with the charge/discharge processes. Figure 4B displays the charge-discharge profiles of the WS_2_/NC composite electrode at current density of 200 mA/g. During the first discharge process, the composite electrode delivered a relatively high specific capacity of about 600 mAh/g, which is attributed from the Na-ion insertion into the WS_2_ and the formation of SEI film. During the following charge process, the electrode showed a reversible capacity of about 375 mAh/g, with the initial coulombic efficiency of 62.5%, meaning irreversible process occurred in the first charge/discharge cycle. It is worth noting that the composite electrode demonstrated similar charge and discharge capacities during the following the cycling, revealing the relatively high stability of the WS_2_/NC electrode for Na^+^ storage.

Rate performances of the pure WS_2_ nanosheets and WS_2_/NC electrodes were tested to demonstrate the enhanced Na^+^ storage performance of the composite electrode. As shown in Figure 4C, the pristine WS_2_ electrode delivered specific capacities of about 358, 320, 287.3, 241.8, 200.3, and 146.7 mAh g^−1^ at current densities of 0.05, 0.1, 0.2, 0.5, 1.0, and 2.0 A g^−1^, respectively. When the current densities were set back to 1.0 and 0.5 A g^−1^, the pristine WS_2_ electrode showed specific capacities of about 192.5 and 236.1 mAh g^−1^ with relatively low reversibility. Interestingly, after N-doped carbon coating, the WS_2_/NC displayed enhanced specific capacities at various current densities, namely 386, 355.1, 326.4, 301.4, 274, and 238.1 mAh g^−1^ at current densities of 0.05, 0.1, 0.2, 0.5, 1.0, and 2.0 A/g, respectively. Also, the composite electrode showed higher reversible capacities of about 271 and 305.5 mAh g^−1^ when the current density went back to 1.0 and 0.5 A g^−1^, respectively. The improved rate performance of WS_2_/NC electrode clearly demonstrates its fast charge transfer during the electrochemical reaction, which can be ascribed to the enhanced electronic conductivity and Na^+^ diffusion velocity resulted from the N-doped carbon coating. Long-term stability of the electrodes was further studied to illustrate the enhanced electrochemical performance of WS_2_/NC for Na^+^ storage. As displayed in Figure 4D, the composite electrode showed an initial discharge capacity of 320.1 mAh g^−1^ and a reversible specific capacity of 290 mAh g^−1^ at current density of 1.0 A/g. For the pure WS_2_ electrode, the initial capacity at 1.0 A/g is the same with that of WS_2_/NC electrode, but the reversible capacity is much lower (only 240.9 mAh g^−1^). After 400 charge-discharge cycles, the composite electrode delivered a capacity of about 180.1 mAh g^−1^, corresponding a loss of 0.09% per cycle, which is much lower than that of the pure WS_2_ with a loss of 0.14% per cycle. The long-term stability comparison further demonstrates the polished Na^+^ storage performance after N-doped carbon coating of WS_2_ electrode.

**Figure 5:**
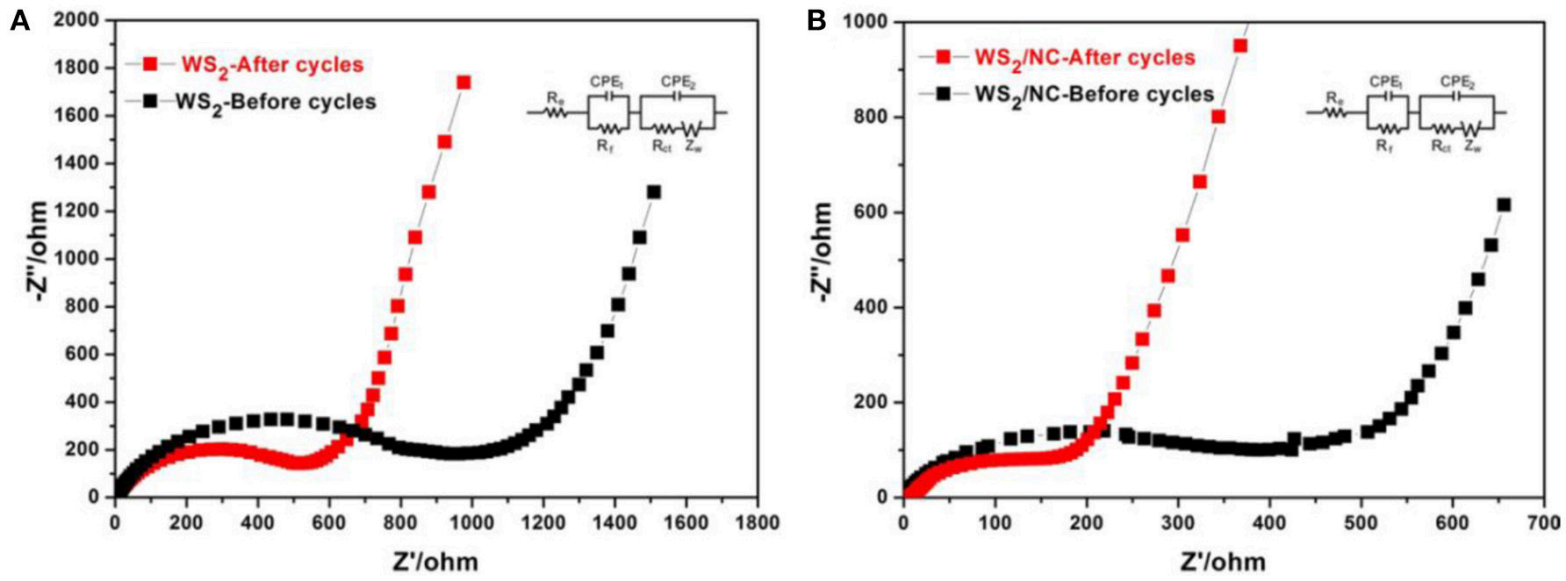
Nyquist plots measured from 0.01 Hz to 1 MHz of **(A)** pure WS_2_ electrode and **(B)** WS_2_/NC before and after 400 cyles, inset shows the EIS test circuitry.

To uncover the reason for the improved electrochemical performance of the WS_2_/NC composite electrode for Na^+^ storage, AC impedance measurements were performed at the initial state and after cycling, as shown in Figure 5. R_e_ is the internal resistance of the as-assembled sodium-ion battery, R_f_ and CPE_1_ are associated with the resistance and constant phase element of the SEI film corresponding to the high-frequency semicircle, R_ct_ and CPE_2_ are corresponding to the charge-transfer resistance and constant phase element of the electrode/electrolyte interface (the semicircle in the medium-frequency region), and Z_W_ represents the Warburg impedance corresponding to the sodium-diffusion process (the inclined line area). The WS_2_/NC composite electrode, at the initial stage, showed a R_ct_ value of about 400 Ω, which is much lower than that of the pristine WS_2_ electrode (about 900 Ω), revealing the faster charge transfer in the composite electrode. Furthermore, after charge-discharge cycling, the R_ct_ values were reduced to 200 and 520 Ω for WS_2_/NC and pure WS_2_ electrodes, respectively, due to the gradual activation and stability of the electrode. Furthermore, the slope of the line in the low-frequency region for WS_2_/NC electrode is much higher than that of the pure WS2 electrode, demonstrating the faster sodium-ion diffusion in the composite electrode during the electrochemically Na+ intercalation/deintercalation process. This fact confirms that the incorporation of N-doped carbon can preserve the high conductivity of the WS_2_/NC composite electrode and greatly enhance rapid electron transport during the electrochemical sodium insertion/extraction reaction, resulting in significant enhancement in the electrochemical performances (Chang and Chen, 2011).

## Conclusions

In summary, WS_2_/NC nanosheets were synthesized and investigated as a high-performance anode for SIBs. It was found that the N-doped carbon layer in the composite electrode contains high content of pyridinic and pyrrolic nitrogen species, which could generate more defect and expose more edge sites in the plane of carbon skeletons, resulting in faster Na^+^ diffusion velocity. Besides, nitrogen doping can efficiently increase the electronic conductivity of the coated carbon layer on the electrode materials, which would decrease the Ohmic polarization and be beneficial for the fast electrochemically sodium ion-storage. As a result, when compared to the pristine WS_2_ electrode, the composite WS_2_/NC electrode showed long-term stability with a loss of 0.09% per cycle and enhanced rate performances. This novel kind of WS_2_/NC composites with high reversible capacity, excellent cyclic stability, and high-rate capability would find wide applications as promising anode materials for SIBs.

## Author contributions

YL, XW, FR, BK, and CY conceived and designed the experiments; HuW, CW, FW, and HaW performed the experiments; HuW, CW, FW, and WZ analyzed the data; HuW and CW wrote the paper; YL, XW, FR and BK revised the paper.

### Conflict of interest statement

The authors declare that the research was conducted in the absence of any commercial or financial relationships that could be construed as a potential conflict of interest.
